# Class IIa bacteriocin resistance in *Enterococcus faecalis *V583: The mannose PTS operon mediates global transcriptional responses

**DOI:** 10.1186/1471-2180-10-224

**Published:** 2010-08-25

**Authors:** Mona Opsata, Ingolf F Nes, Helge Holo

**Affiliations:** 1 Laboratory of Microbial Gene Technology and Food Microbiology, Department of Chemistry, Biotechnology and Food Science, Norwegian University of Life Sciences, N-1432 Ås, Norway; 2 Tine SA, N-0051 Oslo, Norway

## Abstract

**Background:**

The class IIa bacteriocin, pediocin PA-1, has clear potential as food preservative and in the medical field to be used against Gram negative pathogen species as *Enterococcus faecalis *and *Listeria monocytogenes*. Resistance towards class IIa bacteriocins appear in laboratory and characterization of these phenotypes is important for their application. To gain insight into bacteriocin resistance we studied mutants of *E. faecalis *V583 resistant to pediocin PA-1 by use of transcriptomic analyses.

**Results:**

Mutants of *E. faecalis *V583 resistant to pediocin PA-1 were isolated, and their gene expression profiles were analyzed and compared to the wild type using whole-genome microarray. Significantly altered transcription was detected from about 200 genes; most of them encoding proteins involved in energy metabolism and transport. Glycolytic genes were down-regulated in the mutants, but most of the genes showing differential expression were up-regulated. The data indicate that the mutants were relieved from glucose repression and putative catabolic responsive elements (*cre*) could be identified in the upstream regions of 70% of the differentially expressed genes. Bacteriocin resistance was caused by reduced expression of the *mpt *operon encoding the mannose-specific phosphoenolpyruvate:carbohydrate phosphotransferase system (PTS), and the same transcriptional changes were seen in a *mptD*-inactivated mutant. This mutant also had decreased transcription of the whole *mpt *operon, showing that the PTS is involved in its own transcriptional regulation.

**Conclusion:**

Our data confirm the important role of mannose PTS in class IIa bacteriocin sensitivity and we demonstrate its importance involving global carbon catabolite control.

## Background

Bacteriocins are bacterial peptides or proteins inhibitory to bacteria closely related to the producer. Many of the bacteriocins produced by lactic acid bacteria (LAB) have inhibitory spectra spanning beyond the genus level and have a potential in defending unwanted microflora. Since they are produced by food grade bacteria, some are being used in food preservation. However, LAB bacteriocins could have a potential in the medical field. With the increasing spread of antibiotic resistance, the need for alternative antimicrobials is growing. Most of the bacteriocins of LAB are small, heat-stable, cationic peptides and are divided into two classes; class I, the lantibiotics containing modified amino acids and class II, the non-lantibiotics having regular amino acid residues [1]. Among the regular peptide bacteriocins, those belonging to class IIa are produced by a large number of LAB and are best studied [2]. These bacteriocins have highly conserved amino acid sequences, and have a largely common inhibitory spectrum which includes pathogens like *Listeria monocytogenes *and *Enterococcus *spp. Their mode of action is different from common antibiotics [3,4]. Bacterial resistance towards these bacteriocins does not appear to be common in nature [5], while in laboratory experiments resistance to some bacteriocins appear at high frequency [6,7]. Characterization of the resistant phenotype is important for assessment of the usefulness for application of bacteriocins. The target for class IIa bacteriocins is the mannose phosphotransferase system (*mpt*-PTS) [8-11], and mutants lacking a bacteriocin dedicated target are insensitive to the bacteriocin. This mannose PTS is the major uptake system for mannose and glucose in the bacteria [12]. PTS components are also involved in gene regulation of catabolic operons [13]. Hence bacteriocin resistance is likely to cause multiple effects. Among the effects seen in class IIa bacteriocin resistant strains of *L. monocytogenes *are changes in cell envelope, alterations in fatty acid composition [14-17], and a metabolic shift [18]. Enterococci are among the most common LAB habitants in the mammalian microflora, and they are commonly found in fermented foods where they contribute to flavour and preservation, but enterococci have also become the most frequent antibiotic-resistant bacteria in hospitals causing serious infections. As such strains could potentially be defeated by using bacteriocins we need more knowledge about bacteriocin resistance phenomena in enterococci. In this work we have performed transcriptional analyses by genomic microarray to study the effects on class IIa bacteriocin resistance in *E. faecalis *V583, a vancomycin-resistant clinical isolate [19,20]. Our data confirm the important role of the mannose PTS in bacteriocin sensitivity and provide new insight into its role in global gene regulation in this organism.

## Methods

### Bacterial strains and growth conditions

Enterococci were routinely grown at 37°C in M17 (Oxoid) supplemented with 0.5% glucose (GM17) or brain heart infusion (BHI) (Bacto™ BHI, Difco Laboratories, Becton, Dickinson and Company). Growth was monitored using a Bioscreen C instrument (Oy Growth Curves Ab Ltd.), at 37°C.

### Bacteriocin assay

Pediocin PA-1 was obtained from *Pediococcus acidilactici *Pac 1.0 [21] grown for 24 hours in MRS (Oxoid) at 30°C. The culture supernatant was heated to 70°C for 15 min, and applied to a column of SP-sepharose (Amersham Pharmacia Biotech). The column was washed with sodium phosphate buffer (10 mM, pH 5) before the concentrated bacteriocin was eluted with 1 M NaCl. Bacteriocin activity was measured with a 96-well microtiter-plate assay [22]. Stationary phase cultures diluted 100 times in MRS were used as indicators. The plates were incubated for 16 hours at 37°C, and growth was measured spectrophotometrically at 620 nm. One bacteriocin unit (BU) was defined as the amount of bacteriocin that inhibited growth of the indicator strain *E. faecalis *V583 by 50% under these conditions.

### Isolation of resistant mutants

Aliquots from a culture of *E. faecalis *V583 grown in GM17 to an optical density at 600 nm of 1.0 were spread onto GM17 agar plates containing 10 BU/ml pediocin PA-1. After incubation overnight at 37°C, the spontaneously pediocin PA-1 resistant mutant MOP1 was picked. Mutant MOP5 was obtained by inoculating MOP1 in lactic broth [23] supplemented with 800 BU/ml pediocin PA-1. After growth over night the mutant was colony purified on GM17 agar. Mutant MOP2 was resistant to 2-deoxyglucose (2-DG), 2-DG is known to enter the bacteria via mannose PTS [24]. One μl of an *E. faecalis *culture grown overnight at 37°C in M17 broth supplemented with 0.2% fructose was spread onto M17 agar (Oxoid) plates containing 10 mM 2-DG (Sigma) and 0.2% fructose. After incubation for 24 hours, the mutant was isolated. To construct a strain with an inactivated *mpt*, a 355 basepair fragment of gene *mptD *was PCR amplified using primers mptDi-F and mptDi-R and the template was DNA from V583 (Table 1). The fragment was ligated into the *Sna*B1 site in pAS222 [25]. Transformation established the recombination plasmid pGhostΔ*mptD *in *Escherichia coli *EPI300. The resulting plasmid was isolated and electrotransformed into *E. faecalis *V583 as described by Holo and Nes [26]. Transformants were grown at 28°C. Integration into the V583 genome was achieved by growth at 37°C in the presence of tetracycline as described previously [25]. Integration of the plasmid into *mptD *was verified in mutant MOM1 by DNA sequencing using primers mptD-F and mptD-R.

**Table 1:** Plasmids, bacterial strains and primers used in this study

	Description, characteristics^a ^or sequence (5'→3') forward primer, reverse primer	Source or reference
Plasmid		

pAS222	Shuttle vector, TetR	[25]

pGhostΔ*mpD*	Insertion inactivation vector of *mptD*	This work

Strain		

*E. coli *EPI300		Epicentre Technologies, USA

*E. faecalis *V583	Wild type	[20]

MOP1	Resistant mutant, from exposure to pediocin PA-1 10 BU/ml	This work

MOP2	Resistant mutant, from exposure to 10 mM 2-deoxsyglucose	This work

MOP5	Resistant mutant, from exposure to pediocin PA-1 640 BU/ml	This work

MOM1	Inserted inactivated *mptD*	This work

*Pediococcus acidilactici Pac 1.0*	Pedioicn PA-1 producer	[21]

Primer		Target DNA

arcA-F	TAACTCGACAACGGGAAACC	EF0104, *arcA*

arcA-R	TCCCAATGGCCACTACTTCT	EF0104, *arcA*

citE-F	CGGTGATTAACCCTCGTCAA	EF3320, *citE*

citE-R	ACGGAGATAACACCGGAACC	EF3320, *citE*

dnaB-F	TAGAAATGGGGGCAGAATCA	EF0013, *dnaB*

dnaB-R	ATTCGCACGGGACAAACTAC	EF0013, *dnaB*

mptAB-F	TGACCTATGGGGAGGAACAC	EF0020, *mptAB*

mptAB-R	GTCGCAATTTCTTGTGCTGA	EF0020, *mptAB*

mptC-F	ATTCGTATTGCGATTCCAGCA	EF0021, *mptC*

mptC-R	TGCATAACCTACGGCAACGAC	EF0021, *mptC*

mptD-F	TCGTTGGTCATTCATGTGGT	EF0022, *mptD*

mptD-R	GTTGAACTAATGCGGCCAGT	EF0022, *mptD*

mptDi-F	GAAGGAGGAGCAAAGAAAATGGCA	EF0022, *mptD*

mptDi-R	CACCGACACCGGCTAAAGGAC	EF0022, *mptD*

mptO-F	TATCCAAATTCCGTGGGAAG	EF0024, *manO*

mptO-R	TAACACTCGCTTCGGCTCTT	EF0024, *manO*

pgk-F	AATGACGCTCCTTTCCACAC	EF1963, *pgk*

pgk-R	TTTCAAATACGCCCATTGGT	EF1963, *pgk*

^a^TetR, tetracycline resistance

### Metabolites

Glucose, and metabolic products were analyzed by high-performance liquid chromatography and headspace gas chromatography [27,28].

### Acid production

Cells were grown in BHI to OD = 0.2, harvested by centrifugation, then washed and resuspended to the same cell density in 5 mM sodium phosphate buffer pH 6.9 containing 0.025% bromocresol purple. Acidification was monitored at 37°C in 200 μl reaction volumes in microtiter plates using a microtiter reader recording absorbance at 620 nm after the addition of either glucose or glycerol (1%).

### RNA isolation, cDNA synthesis and microarray experiments

Cultures of strain V583 and its mutants grown overnight in (BHI) (Bacto™ BHI, Difco Laboratories, Becton, Dickinson and Company) were diluted 1:50 in BHI and incubated further. Bacterial cells were harvested at OD 600 nm 0.2 by centrifugation, washed in TE-buffer (10 mM Tris-HCl, 1 mM EDTA pH 7.4), and quickly frozen in liquid nitrogen. From the frozen bacteria pellets (-80°C) lysate was obtained after lysozyme digestion (10 μg/ml) and total RNA was extracted using the RNeasy Mini kit (Qiagen) according to the manufacturer's protocol. Residual DNA was removed on-column with RNase free DNase (Qiagen) (27 Kunitz units). The integrity of RNA samples was verified using capillary electrophoresis on prokaryotic total RNA Nano LabChip with Bioanalyzer 2100 (Agilent Technologies), and purity and concentration were determined by optical density measurements with NanoDrop ND-1000 (NanoDrop Technologies, Inc.). Synthesis of cDNA and incorporation of aminoallyl-labeled dUTP (Sigma) were performed at 42°C for 3 hours with Superscript III (Invitrogen) after preheating 10 μg of total RNA with 30 μg random hexamers as specified by Aakra et al. [29]. RNA in the cDNA samples was hydrolyzed and then the reactions were neutralized [29]. The cDNA was purified by washing through MinElute columns (Qiagen) and dried in a vacuum centrifuge. Coupling of the aminoallyl-labelled cDNAs to the fluorescent *N*-hydroxysuccinimide-ester dyes; cyanine-3 and cyanine-5 (in DMSO) (Amersham Pharmacia) were done for 30 min in 18 μl 50 mM Na_2_CO_3 _buffer pH 9.3. The probe was purified through MinElute columns and dried. Corresponding probes generated from the wild type and the mutant samples were combined, then prehybridisation, hybridisation, washing and drying were performed as described [29]. Scanning of hybridized microarray slides were done with Agilent G2505B scanner (Agilent Technologies). Transcriptome analyses were performed using whole-genome DNA microarray of the *E. faecalis *V583 genome containing PCR fragments representing 94.7% or 3160 of all open reading fragments in five copies on each slides [29].

### Data analysis

The microarray images were analyzed using GenePix Pro 6.0 software (Axon), and raw data from each slide was preprocessed independently. The images were gridded to locate the spots corresponding to each gene. Fluorescence intensities for mean spot signal to median background from both channels (532 nm, Cy3 and 635 nm, Cy5) were extracted for data analysis and normalization. Spots with diameter <60 micrometer and spots of low quality were filtered. All filtering and Lowess normalization were performed in BASE (BioArray Software Environment) [30]. Average log2-transformed intensity Cy3/Cy5 ratio for each gene in 5 replicates on each array was calculated. Statistical analyses using SAM (Significance Analysis of Microarrays) were performed on the normalized microarray data to identify significant differentially expressed genes in each of the individual mutants by one-class analyses [31]. SAM was used with a stringent confidence level by setting the false discovery rate, FDR, to zero, meaning no genes were identified by chance. The microarray data obtained in this study has been deposited in the ArrayExpress database (http://www.ebi.ac.uk/arrayexpress/) with accession number E-TABM-934.

### Quantitative real-time PCR (qPCR)

RNA was isolated independently from that used for transcriptome analysis. Synthesis of cDNA were performed from 150 ng of total RNA confirmed free of DNA after an additional DNase treatment, 6 μg hexamers, 10 mM of dNTP with Superscript III and supplied reagents as described above. The primers used in real-time quantitative PCR are listed in Table 1. Real-time PCR was performed with a cDNA dilution in triplicates, representing 0.75 ng RNA, 0.1 μM of each primer with FastStart SYBR Green master included ROX (Roche Applied Science) on an ABI Prism 7700 Sequence Detection System (Applied Biosystems). After denaturation at 95°C the program was 40 cycles, including 95°C for 15 seconds, 30 seconds at 62°C and 72°C for 30 seconds. Standard curves were made for each primer pair to calculate amplification efficiency of the target genes and the endogenous control gene (EF0013). Differential expression was determined by calculating the change in threshold cycles for each gene with the ΔΔCt-method, with RNA isolated from resistant mutants and wild type bacteria.

### DNA manipulations and sequencing

Isolation of DNA from *E. faecalis *V583 and mutants was done using Advamax-beads (Advanced Genetic Technologies Corp.). PCR products were generated with Phusion DNA polymerase (Finnzymes). Other enzymes for DNA manipulation were from New England Biolabs. DNA fragments were purified by use of agarose gel electrophoresis and Qiaquick PCR purification columns (Qiagen). Plasmids were isolated using Qiagen miniprep columns. Standard procedures [32] were used for restriction cutting of DNA, ligation and cloning in *E. coli*. DNA was sequenced using the ABI Prism BigDye terminator sequencing ready reaction kit version 3.1 and analyzed with the ABI Prism 3100 genetic analyzer according to the supplier's procedures (Applied Biosystems).

## Results

### Isolation and characterization of bacteriocin resistant mutants

**Figure 1:**
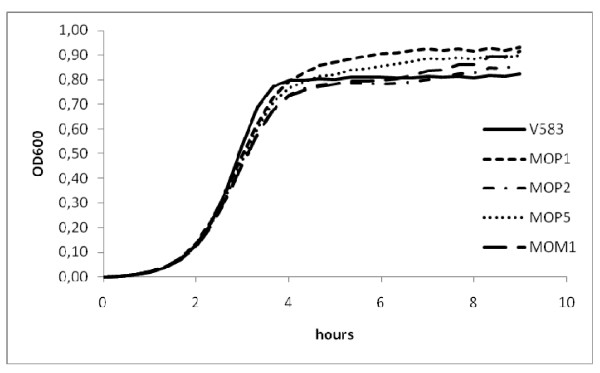
**Growth curves of *E. faecalis *V583 and the resistant mutants in BHI**. Each graph is based on average of ten parallels.

Four class IIa bacteriocin resistant mutants of *E. faecalis *V583 were obtained. Mutants MOP1 and MOP5 were isolated after exposure to two different concentrations of pediocin PA-1. A third spontaneous mutant (MOP2) was obtained by selecting colonies resistant to 2-DG. The MOP2 mutant was also resistant to pediocin (Table 2). Pediocin PA-1 resistant mutants were isolated at a frequency of 3 10^-4^, consistent with reported resistance frequency in *Enterococcus *and *Listeria *[6,7]. Previous studies have shown that pediocin resistance can be obtained by mutations in the mannose PTS operon, *mpt *[33,34], therefore we constructed a resistant *E. faecalis *V583 (MOM1) disrupted in *mptD*. Mutants MOM1 and MOP5 were highly resistant to pediocin PA-1, while MOP1 and MOP2 were less resistant (Table 2). The pediocin resistance phenotype was stably maintained in all mutants in the absence of bacteriocin. All mutants were resistant to 2-DG (results not shown). In exponential phase up to an optical density of 0.2 the mutants grew with the same growth rate as the wild type, after which they grew slower but reached a somewhat higher final density than the wild type (Figure 1).

**Table 2:** Sensitivity to pediocin PA-1 of strains used

Strain	MIC (BU/ml)
V585	5

MOP1	160

MOP5	>21·10^6^

MOP2	160

MOM1	>21·10^6^

The mutant strains showed reduced glucose consumption during growth (Table 3). In addition, these mutants displayed changes in the metabolic profile by producing less lactate than the wild type, but more formate and ethanol.

**Table 3:** Metabolites in supernatants of BHI-grown *E. faecalis *V583 and mutant strains

		Metabolites (mM)					
		
Strain or genotype	OD600 nm	Glucose	Citrate	Lactate	Formate	Acetate	Ethanol
V583	0.2	1.95	0.19	6.13	0.02	1.30	0.44
MOM1	0.2	1.03	0.21	3.64	0	1.30	0.41
MOP1	0.2	1.01	0.00	1.03	0.05	1.38	0.60
V583	0.8	7.82	1.02	20.76	5.30	7.14	3.28
MOM1	0.85	7.82	1.02	17.40	16.44	8.82	5.61
MOP1	0.9	7.82	1.02	11.60	18.79	10.74	8.72

The composition of the BHI growth medium was: glucose, 7.82 mM; citrate 1.02 mM; acetate 4.50 mM; formate 0.01 mM, and other substrates in low concentrations.

**Figure 2:**
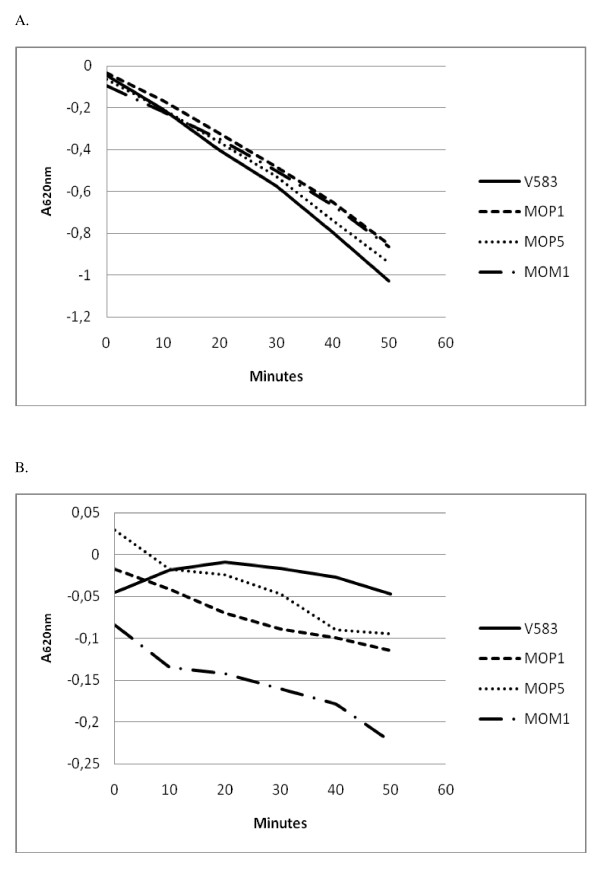
**Acid production from glucose (A) and glycerol (B) by cell suspensions of *E. faecalis *V583 and resistant strains**. Each graph is based on average of three parallels.

Acid production was measured using washed cell suspensions with glucose or glycerol as substrates (Figure 2). The wild type produced acid from glucose more rapidly than the mutants. Acid production from glycerol was faster in the mutants. However, the rates were much lower than with glucose, and the wild type did not show detectable acid production.

### Transcriptional analyses of pediocin resistant strains of *E. faecalis *V583

The transcriptional profiles of each of the four pediocin resistant mutants were compared to that of the parent strain using DNA microarrays of *E. faecalis *V583 under standard growth conditions. The microarray data used are the means of four independent biological replicates for the spontaneous mutants and four replicates for the *mptD*-inactivated mutant. Significant differentially expressed genes in each of the individual mutants were identified using one-class analysis in the statistical software SAM [31]. The three spontaneous mutants showed large similarities in transcriptional responses, and by using the two-class module in SAM no significant difference between them could be identified. Furthermore, DNA sequencing showed no mutations in the *mpt *operon in any of these mutants, but they all had the same transversion mutation in EF0018 resulting in amino acid substitution A356G in the transcription regulator MptR. This alanine is conserved among MptR homologs (results not shown). Consequently, to gain strength to the statistical analysis all the 12 microarrays representing the spontaneous mutants were treated as parallels of the same experiment and called MOP. In MOP 119 genes showed more than two-fold change in expression, and in MOM1 184 genes were differentially expressed. Most of the genes were upregulated; only 15 and 11 genes were downregulated in MOP and MOM1, respectively. These genes and their expression profiles are listed in Additional file 1. As shown in Additional file 1, MOP and MOM1 had very similar transcriptional profile, but we observed enhanced fold change ratio of nearly every gene in the *mptD*-inactivated mutant compared with the spontaneous mutants. Two-class analysis identified 24 genes with a significant difference in transcription between MOM1 and MOP, and 12 of them had more than two-fold change in expression in the Δ*mptD *mutant only (Table 4).

**Table 4:** Genes identified with significant different transcriptional profile between MOM1 and MOP mutants of *E. faecalis*

ORF	**Log**_**2 **_**ratio MOP**	**Log**_**2 **_**ratio MOM1**	Protein encoded by gene (Gene name)
EF0071	-0.37	0.77	lipoprotein, putative

EF0352	-0.15	-0.75	hypothetical protein

EF0751	0.63	-0.51	conserved hypothetical protein

EF0754	0.25	-0.68	conserved hypothetical protein

EF0755	-0.03	-1.35	conserved hypothetical protein

EF0900	0.19	2.00	aldehyde-alcohol dehydrogenase (*adhE*)

EF1036	0.49	2.76	nucleoside diphosphate kinase

EF1227	-0.01	1.06	conserved hypothetical protein

EF1422	0.11	0.85	transcriptional regulator, Cro/CI family

EF1566	-0.64	0.57	3-phosphoshikimate 1-carboxyvinyltransferase (*aroA*)

EF1567	-0.39	0.52	shikimate kinase (*aroK*)

EF1603	-0.15	1.01	sucrose-6-phosphate dehydrogenase (*scrB-1*)

EF1619	-0.33	2.31	carbon dioxide concentrating mechanism protein CcmL, putative

EF1624	-0.38	1.58	aldehyde dehydrogenase, putative

EF1627	-0.36	2.79	ethanolamine ammonia-lyase small subunit (*eutC*)

EF1629	-0.24	2.27	ethanolamine ammonia-lyase large subunit (*eutB*)

EF1732	0.37	2.01	ABC transporter, ATP-binding/permease protein, MDR family

EF1750	-0.04	0.46	endo/excinuclease amino terminal domain protein

EF1760	0.11	0.48	cell division ABC transporter, permease protein FtsX, putative

EF1769	0.01	1.45	PTS system, IIB component, putative

EF2216	0.07	0.80	hypothetical protein

EF2254	-0.06	-1.37	hypothetical protein

EF2887	0.26	-0.40	Not annotated

EF3029	0.14	0.64	PTS system, IID component

EF3041	0.07	-0.58	pheromone binding protein

The genes were identified by two-class SAM analyzes and their corresponding expression levels are included.

**Figure 3:**
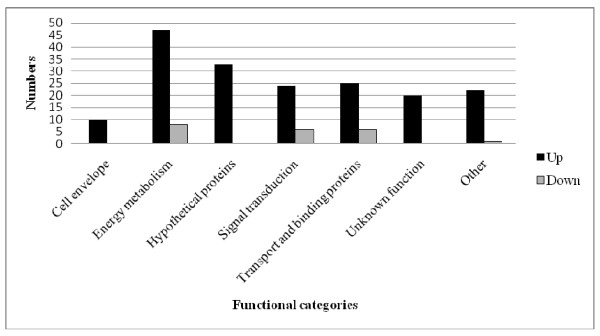
**Numbers and functional categories of the 207 genes differentially expressed in resistant strains of *E. faecalis *V583**.

The differentially expressed genes are distributed across the entire genome and the majority encodes proteins involved in energy metabolism, transport and binding, signal transduction, or of unknown functions (Figure 3). Validation of the differential expression of nine genes was performed using quantitative real-time PCR (qPCR). These genes represented different patterns of expression from various functional groups. As shown in Table 5, the results were in general in high concordance with the microarray results but the strongest responses were more pronounced with qPCR, demonstrating the wider dynamic range of response by this technique.

**Table 5:** Gene expression analyzed by quantitative real-time PCR and microarray of mutant MOP1

		Microarray	Quantitative RT-PCR
**Gene ID**	**Gene**	**Log**_**2 **_**ratio**	**Log**_**2 **_**ratio**

EF0013	*dnaB*	0.02	0.04

EF0020	*mptAB*	-2.80	-2.07

EF0021	*mptC*	-0.68	-3.07

EF0022	*mptD*	-1.70	-2.48

EF0024	*manO*	-0.59	-3.29

EF0105	*argF-1*	3.06	3.83

EF0106	*araC*	3.02	3.28

EF0633	*tyrS-1*	-0.82	-1.46

EF1963	*pgk*	-1.53	-2.71

EF3320	*citE*	4.90	5.83

Gene regulation values (log_2_) are the average results of four biological replicates for microarray experiments and of two biological replicates for quantitative PT-PCR.

### Genes showing reduced expression in bacteriocin resistant mutants

**Figure 4:**
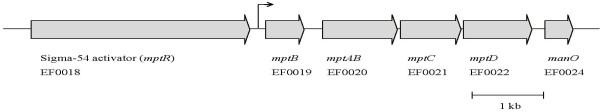
**Genetic organization of the mannose PTS operon of *E. faecalis *V583 and the preceding σ**^**54**^**-associated activator gene *mptR***. The *mpt *operon contains the *mpt *genes, an additional gene encoding an EIIB and the distal gene that resembles *manO*. The σ^54^-promoter sequence is indicated by an arrow.

Only few genes were significantly downregulated in the resistant mutants. These genes encode proteins involved in transport, binding and energy metabolism. Most pronounced effects in transcription of the pediocin resistant mutants was the strong reduction in gene expression of the mannose PTS operon (EF0019-EF0022). This *mpt *operon is σ^54^-regulated [34], and has an unusual gene organization as it contains an additional gene encoding a distinct EIIB in front of the genes for the EIIAB, EIIC and EIID proteins and the last gene EF0024 (Figure 4). Despite the strong down-regulation, the signals were not completely abolished. Quantitative real-time PCR analyses confirmed reduced transcription of *mptC *representing the *mpt *operon (Table 5). The downstream gene EF0024 was also downregulated indicating that it belongs to the *mpt *operon. This gene, referred to as *manO *[35], encodes a protein highly conserved among strains of lactic acid bacteria, is part of the mannose PTS operon in *L. monocytogenes *and *Lactobacillus casei *[36,37].

As expected, MOM1 showed reduced hybridization to the *mptD *probe, but the mutant also exhibited reduced expression of the upstream genes in the *mpt *operon indicating that MptD is involved in the regulation of its own synthesis. Strongly reduced gene expression of EF0082 encoding a major facilitator family transporter was detected in both the spontaneous mutants and in the Δ*mptD *mutant. Interestingly, also the genes *gap-2*, *pgk*, *triA*, *eno *(EF1961-64), *gpm *(EF0195), *pyk *(EF1046) and *ldh-1 *(EF0255) encoding enzymes of glycolytic metabolism were expressed to a lower extent in the resistant strains. Of the remaining genes for the complete pathway for glucose consumption, *fba *and *pfk *showed 1.6-fold reduced expression (excluded by the 2-fold-change cut off in Additional file 1). Furthermore, the genes in the fructose operon encoding a transcription regulator, fructose-specific PTS IIABC and 1-phosphofructokinase (*fruK-2*), showed reduced transcription in all mutants.

### Genes with enhanced expression in bacteriocin resistant mutants are involved in alternative pathways of energy metabolism

About 90% of the differentially expressed genes were upregulated in the mutants, and of them having an ascribed function most encode proteins involved in energy metabolism, transport, binding and signal transduction (see Additional file 1).

Since the *mpt *operon is σ^54^-regulated, we examined if other σ^54^-controlled genes were affected in the mutants. By *in silico *analysis of the genome sequence of *E. faecalis *V583 using the sigma-54 promoter specific consensus sequence of *B. subtilis *YTGGCACNNNNNTTGCW [38], 10 putative σ^54^-dependent promoters were identified. Four of them are preceded by a gene encoding a σ^54^-dependent activator, and downstream genes encoding PTS enzyme II. Only the *mpt *operon showed reduced expression, while up-regulation only was observed for *mphD *localized downstream of EF1955 encoding a LevR-like σ^54^-dependent activator.

### Involvement of catabolite-responsive elements (*cre*)

The large number of up-regulated catabolic genes in the mutants suggests the involvement of a global regulator. In Firmicutes carbon catabolite repression (CCR) is mediated via binding of the catabolite control protein A (CcpA) to operators known as catabolite-responsive elements *cre *[39]. By searching the *E. faecalis *V583 genome using the *cre *query consensus sequence WTGNAANCGNWNNCW developed for *B. subtilis *[40], we found 34 intergenic putative catabolite-responsive elements, and 21 of them were in the promoter regions of operons showing significant increased transcription in the mutants (see Additional file 1). Another 42 of the promoter regions of differentially expressed genes contained sequences with one mismatch to the *B. subtilis cre*-consensus. We propose that these sequences represent *cre-*sites of *E. faecalis *(see Additional file 2). Their sequences were aligned and had the consensus sequence WTGWAARCGYWWWCW. Many of the differentially expressed genes contained this sequence in their coding regions, and two were located in the intergenic regions downstream the down-regulated genes EF0635 and EF1046 (see Additional file 1). As shown in Additional file 1, a large majority of the differentially expressed genes are associated with putative *cre*-sites, and seven of them possibly regulate divergent expression. Many of the up-regulated genes located downstream of putative *cre-*sites encode enzymes involved in the use of alternative energy and carbon sources. Among them, genes encoding enzymes involved in citrate transport and catabolism (EF3314 to 3328) had the greatest increase in expression, up to sixty-fourfold in the mutants. A *cre*-site was found in the intergenic region between the two divergent *cit *operons. The *arc *operon, preceded by a *cre*-site encodes the energy yielding enzymes by arginine consumption, was also up-regulated in the mutants. Other substrates whose catabolic genes appear to be repressed via *cre*-sites in the wild type and not in the mutants were serine, galactose, lactose, glycerol and sucrose.

The transcription of several transcriptional regulators appeared to be regulated via *cre*-sites, suggesting involvement of CCR in regulatory cascades. None of the genes encoding proteins mediating CCR (*hpr*K, *ptsH *and *ccpA*) had significantly changed expression.

Ten of the genes showing enhanced expression encode proteins predicted to contribute to virulence [19]; proteins involved in chitin catabolism (EF0361 + 62), polysaccharide lyase (EF0818), serine protease and coccolysin (EF1817 + 18), secreted lipase (EF3060), two ABC transporters of iron and peptides (EF3082, EF3106), lipoprotein of YaeC family (EF3198), and cell surface anchor family protein (EF3314). All of them were associated with *cre*-sites and therefore under potential CCR regulation.

## Discussion

We compared the transcriptomes of wild type *E. faecalis *V583 and stable pediocin PA-1 resistant mutants. The mutants were spontaneously resistant isolates, and since sensitivity to class IIa bacteriocins in *E. faecalis *is dependent on *mpt*, we also constructed and studied an insertion inactivated *mptD *mutant. The transcriptomes were obtained from cells grown to early exponential growth phase in rich medium.

In *E. faecalis *the *mpt *operon is under transcriptional control from a promoter recognized by σ^54 ^and depending on the activator MptR, encoded by EF0018 [33,34]. The spontaneous bacteriocin resistant isolates contain a mutation in *mptR *causing down-regulation of the *mpt *operon. Mutant MOP5, derived from MOP1, was resistant to higher bacteriocin concentrations than the other spontaneous mutants, but we could not identify sequence differences in *mptR *or the *mpt *operon between these mutants, indicating that changes in other DNA sequences may also contribute to bacteriocin resistance in *E. faecalis*.

Our data confirm previous findings on the role of the mannose PTS in bacteriocin sensitivity, but the most striking results were the extensive changes of transcription among genes involved in carbohydrate metabolism, caused by inactivation of the *mpt *PTS. The mutants showed reduced glucose consumption, demonstrating the important role of Mpt in glucose metabolism in *E. faecalis*. Glucose consumption was not abolished, however, showing that the bacteria have alternative, less efficient glucose uptake systems, probably among the transport systems upregulated in the mutants. The presence of multiple glucose uptake systems is common in bacteria, and transporters additional to the mannose PTS were recently described in *Lactococcus lactis *and *L. monocytogenes *[41,42]. Impaired glucose uptake and metabolism affects the energy status of the cells, leading to changes in concentrations of glycolytic metabolites. Yebra et al [37] showed that disruption of the mannose PTS caused a slower glucose uptake and relief of glucose repression in *L. casei*.

The altered energy status is sensed by the HPr-kinase/phosphorylase and implemented on the PTS phosphorcarrier protein HPr [13,43-45]. In cells with a high energy status HPr is phoshorylated at Ser-46 and can then form a complex with CcpA [43,45]. In CCR or CCA (carbon catabolite activation) the CcpA/HPr-Ser-P complex regulates transcription through binding to the *cre-*sites [46].

Most of the differential gene expression observed in our experiments could be ascribed to carbon catabolite regulation via *cre-*sites. CCR in *E. faecalis *has been studied by others, but not by transcriptomic analysis. It has been reported that enzymes for degradation of citrate, arginine, serine, galactose and glycerol are under control of CCR in *E. faecalis *[47-50]. This is in agreement with our finding that these genes are up-regulated and associated with *cre*-sites. The metabolism of glycerol shows that our mutants were catabolic derepressed.

The consensus sequence of the extragenic putative *cre*-sites compiled in this study is WTGWAARCGYWWWC, very similar to what has been reported in *B. subtilis *[40]. Most of the operons affected contain upstream *cre*-sites, but in several cases the putative *cre*-site is found within the open reading frames. Interestingly, three of the differentially expressed genes have the putative *cre*-site positioned in the intergenic region immediately downstream of the genes. Regulation of transcriptional initiation involving a 3'-*cre *located within the open reading frame but distantly separated from the promoter has been suggested to involve DNA looping [51]. To our knowledge, *cre*s located downstream of the regulated gene have not been reported.

Another down-regulated gene with a putative *cre*-site in its promoter was EF0082, encoding a major facilitator family transporter. The gene has also been found to be positively regulated by a PrfA-like regulator, Ers, encoded by EF0074 [52].

Altogether, transcription involving about 90 *cre*-sites appeared to be affected in *E. faecalis *by disturbing its mannose PTS. About 65% of the putatively CCR regulated genes encode proteins involved in uptake and metabolism of alternative energy sources. It is noteworthy that a number of genes showing increased transcription in our mutants encode transcription regulators suggesting that regulatory cascades are involved. Among them were EF1025 and EF1026, encoding the homologs of CcpN and Yqfl which are involved in CcpA independent CCR in *B. subtilis *[53].

When phosphorylated at His-15 by phosphotransfer from phosphoenolpyruvate via enzyme I, HPr has other regulatory functions. HPr-His-P reaches high levels in cells with a low energy status in response to reduced levels of glycolytic intermediates and ATP, and increased level of Pi and PEP [12]. It can by phosphorylation regulate the activity of PTSs, enzymes such as DhaK and GlpK and transcriptional regulators [13,48,54,55].

Interestingly, not only the spontaneous mutants but also the *mptD*-inactivated mutant showed a strong reduced transcription of the *mpt *operon. The decreased transcription was also seen in the genes upstream of the disrupted *mptD*, suggesting that a functional PTS is necessary for transcription of the *mpt *operon. The *mpt *regulator MptR contains two PTS regulatory domains (PRDs) flanking an EIIA domain like its homologs, ManR of *Listeria innocua *and the well studied LevR of *B. subtilis *[13,56,57]. Phosphorylation in EIIA of LevR mediated by HPr-His-P leads to activation of *lev *transcription, while phosphorylation of PRD-II at His-869 by the specific PTS EIIB^Lev ^negatively regulates transcription. Based on mutation analyses it was suggested that *mpt *transcription in *L. innocua *is similarity regulated by phosphorylation of ManR, and that phosphorylation at both sites would also downregulate *mpt *transcription [58]. Such a model can be reconciled with our findings on *mpt *transcription regulation in *E. faecalis*, and in the *mptD-*inactivated mutant EIIAB^Mpt ^will phosphorylate MptR (at PRD-II) and thereby negatively regulate transcription of its own operon. We cannot exclude that the weak *mpt *signals of MOM1 are caused by altered mRNA stability. Reduced expression was also seen for EF0024 located downstream of *mptD*, indicating it being a part of the *mpt *operon. This gene is highly conserved downstream the mannose PTS genes in lactic acid bacteria, *Listeria *and *Clostridium*, and it is down-regulated in a σ^54^-mutant of *L. monocytogenes*, implying that it is part of the mannose PTS operon also in this organism [36].

The *mph *operon is regulated by another σ^54^-depending regulator, encoded by EF1955 [34], which has a domain architecture similar to MptR and LevR and the phosphorylatable histidines are conserved among the three regulators. The up-regulation of the *mph *operon seen in our mutants can probably be ascribed to activation of the regulator by phosphorylation of its EIIA^Mph^-domain (His-566) by HPr-His-P. Such activation would be prevented in the wild type growing on glucose [13].

HPr-His-P can control transcription dependent on regulators containing PTS domains and PRDs [13]. Two PRD containing antiterminator proteins were identified in the *E. faecalis *genome, and enhanced expressions was observed for one (EF1515), along with the downstream gene encoding an N-acetylglucosamine-specific EIIABC, a multidomain PTS protein. Regulators of this BigG-family cause release of termination structures in mRNA and enhanced transcription of downstream PTS genes when activated by HPr-His-P [59,60], which can explain the increased gene expression in the mutants. In an analogous manner, the increased expression seen for the ascorbate-specific EIIB and EIIC genes are possibly caused by HPr-His-P mediated phosphorylation of the regulator encoded by the upstream EF2966. The EF2966 gene product contains PRDs and PTS domains and is probably a transcription regulator, but has erroneously been annotated as a BglG-type antiterminator although it lacks an RNA-binding domain [55].

Possibly, most or all the changes in gene expression in the mutants are caused by altered energy status of the cells, sensed by changes in metabolite concentrations. But not all the effects seen in our mutants could be directly ascribed to HPr phosphorylation. In *E. faecalis *fructose utilization is not under CCR [50,61], and no *cre*-site was detected in the *fru *promoter region of the downregulated *fru *operon (EF0717-19). This is in contrast to *L. lactis *where fructose utilization is regulated via CCR [62]. The fructose operon in *L. lactis *is also regulated by FruR and activation is dependent on fructose-1-phosphate [62]. The *fru *operon (EF0717-19) has a similar genetic organization in *E. faecalis*, including a *fruR *homolog and a putative FruR recognizing promoter which suggests that the *fru *operon is under repression of FruR in the mutants due to lowered intracellular levels of fructose-1-phosphate.

All the genes encoding enzymes leading from glucoses to lactic acid were down-regulated in the mutants. The *ldh-1*, encoding the major lactate dehydrogenase in *E. faecalis *[25], appears to be regulated by CCA, like in *L. lactis *[63]. Genes in the central glycolytic operon (*gap-2*, *pgk*, *tpiA*, *eno*) showed reduced expression probably as a consequence of low fructose-1,6-bis phosphate (FBP) concentration, and repression mediated by the central glycolytic gene repressor CggR encoded by the first gene in the operon, EF1965. A putative CggR operator sequence upstream of EF1965 was identified using the criteria of Doan & Aymerich [64]. In *B. subtilis*, the repressor binds the operator localized upstream of *cggR *when not bound to FBP [64,65].

The observed shift in metabolic profile toward more mixed acid fermentation reflects the transcriptional changes observed, but also the changes in concentration of central metabolic intermediates [66].

The spontaneous mutants MOP1 and MOP2 showed some Mpt activity, as substantiated by intermediate bacteriocin sensitivity. The deletion mutant could not have any Mpt activity and would probably have a lower energy status than the other strains. In agreement with this, we observed quantitative differences in responses between the spontaneous mutants and the constructed mutant. Generally, all transcriptional effects were stronger in the constructed mutant. In *B. subtilis *Singh and colleagues [67] reported that the strength of *cre*-site dependent CCR is dependent only of the HPr-Ser-P levels in the cells, with involvement of different co-repressors as glucose-6-P and FBP [68]. We show that difference in strength of CCR is not only limited to *cre*-site dependent CCR.

Abranches et al [69] studied the transcriptome of an EIIAB mannose-PTS mutant of *S. mutans*. A much lower number of genes were upregulated in that case, but largely the effects were similar to our results of *E. faecalis*. Like in the pediocin resistant *E. faecalis*, a significant number of genes encoding uptake systems and catabolic enzymes were up-regulated, demonstrating its central role in regulation of energy metabolism in these organisms. However, notable differences were also seen; PTS for trehalose (EF0958) and a gene for sodium-iron-translocating uptake (EF3324), both downregulated in the *S. mutans *mutant were up regulated in the *E. faecalis *mutants. Moreover, central glycolytic genes showed an opposite regulation in the two species. These differences could be a result of niche adaptation and reflect the difference in habitat of these human lactic acid bacteria. The fitness cost associated by a lack of CCR is a probable reason why mutants resistant to class IIa bacteriocins are rarely isolated from nature.

## Conclusion

We have demonstrated global transcriptional effects in *E. faecalis *mutants resistant to class IIa bacteriocins, caused by changes in the *mpt *operon. The majority of the effects can be attributed to relief from glucose repression and lack of CCA. This mannose PTS is central in regulating carbon catabolite control in this organism. Our study is the first to characterize the *cre*-dependent and -independent responses in carbon catabolite control in enterococci.

## Authors' contributions

MO participated in the design of the study, carried out the experimental work, image and statistical analyzes, analyzed and interpreted data, and wrote the manuscript. HH conceived the study, participated in the design of the study and data interpretation, and helped to write the manuscript. IFN conceived the study, participated in the design of the study and corrected the manuscript. All authors have read and approved the manuscript.

## Supplementary Material

Additional file 1 **Table A1: Transcriptional differences between the bacteriocin resistant mutants and the wild type**. ^a^The gene expression ratios are shown as the log2 values of expression in the mutant samples, MOP and MOM1, over that in the wild type, of the differentially expressed genes. Gene expression ratio are indicated by 1 when the fold-change ration data are under 2 and/or the q-values are higher than 0. ^b^Gene included with special interest, when not meet the statistical thresholds. ^c^Putative *cre*-site adjacent gene is indicated with an arrow and illustrates gene(s) controlled by the same *cre*-site. The arrow is solid filled when the *cre*-site corresponds to the *cre*-consensus proposed by Miwa [40], and the arrow is not filled when it contains one mismatch. The *cre*-site position is either localized in the promoter^a^, intragenic^b ^or downstream of the gene (gradient filled arrow). ^d^The functional categories are: A. Amino acid biosynthesis, B. Biosynthesis of cofactors, prosthetic groups and carriers, C. Cell envelope, D. Cellular processes, E. Central intermediary metabolism, F. DNA metabolism, G. Energy metabolism, H. Hypothetical proteins, I. Protein fate and synthesis, J. Purines/pyrimidines/nucleosides/nucleotides, K. Regulatory functions, L. Signal transduction, M. Transcription, N. Transport and binding proteins, and O. Unknown function. Click here for file

Additional file 2 **Table A2: Summary of the putative *cre*-sites of regulated genes in the mutant strains**. Sequence and start position of the 63 putative promoter catabolite-responsive elements of the regulated genes in the pediocin PA-1 resistant mutants, MOM1 and MOP of *E. faecalis *V583. Click here for file
