# Supplementary material for: Class IIa bacteriocin resistance in Enterococcus faecalis V583: The mannose PTS operon mediates global transcriptional responses
Source: BMC Microbiol. 2010 Aug 25;10:224. doi: 10.1186/1471-2180-10-224 (PMC2941500; doi:10.1186/1471-2180-10-224)
Supplement: Additional file 1 — Table A1: Transcriptional differences between the bacteriocin resistant mutants and the wild type. aThe gene expression ratios are shown as the log2 values of expression in the mutant samples, MOP and MOM1, over that in the wild type, of the differentially expressed genes. Gene expression ratio are indicated by 1 when the fold-change ration data are under 2 and/or the q-values are higher than 0. bGene included with special interest, when not meet the statistical thresholds. cPutative cre-site adjacent gene is indicated with an arrow and illustrates gene(s) controlled by the same cre-site. The arrow is solid filled when the cre-site corresponds to the cre-consensus proposed by Miwa [40], and the arrow is not filled when it contains one mismatch. The cre-site position is either localized in the promotera, intragenicb or downstream of the gene (gradient filled arrow). dThe functional categories are: A. Amino acid biosynthesis, B. Biosynthesis of cofactors, prosthetic groups and carriers, C. Cell envelope, D. Cellular processes, E. Central intermediary metabolism, F. DNA metabolism, G. Energy metabolism, H. Hypothetical proteins, I. Protein fate and synthesis, J. Purines/pyrimidines/nucleosides/nucleotides, K. Regulatory functions, L. Signal transduction, M. Transcription, N. Transport and binding proteins, and O. Unknown function. [file 1471-2180-10-224-S1.PDF]

ORF	Putative promoter *cre* -site[c]	Putative intragenic *cre* -site[c]	Expression ratio MOP[a]	Expression ratio MOM1[a]	Functional categories[d]	Protein encoded by gene and gene name
Up-regulated						
EF0052			1.21	1	H.	hypothetical protein
EF0053			1.64	2.47	F.	DNA polymerase III, epsilon subunit (*dnaQ*)
EF0054			1	1.54	H.	hypothetical protein
EF0069			1.94	2.52	E.	N-acetylmannosamine-6-phosphate epimerase
EF0077			1.02	1	H.	conserved hypothetical protein
EF0097			1.42	1.34	D.	regulatory protein pfoR, putative
EF0098			1.40	1.45	G.	L-serine dehydratase, iron-sulfur-dependent, beta subunit (*sdhB-1*)
EF0099			1.57	2.21	G.	L-serine dehydratase, iron-sulfur-dependent, alpha subunit (*sdhA-1*)
EF0100			1.30	1.62	I.	seryl-tRNA synthetase (*serS-1*)
EF0103			1.38	1	K.	transcriptional regulator, ArgR family
EF0104			2.92	1.67	G.	arginine deiminase (*arcA*)
EF0105			3.06	2.21	G.	ornithine carbamoyltransferase (*argF-1*)
EF0106			3.02	2.77	G.	carbamate kinase (*arcC-1*)
EF0108			2.89	2.42	N.	C4-dicarboxylate transporter, putative
EF0114			1.98	2.68	C.	glycosyl hydrolase family 20 protein, beta-hexosaminidase
EF0115			2.45	2.25	M.	endoribonuclease L-PSP, putative
EF0173			1	1.07	J.	pyrimidine-nucleoside phosphorylase (*pyn*)
EF0253			1.00	1	G.	aldehyde dehydrogenase[b]
EF0270			1	1.21	L.	PTS system, beta-glucoside-specific IIABC components
EF0271			1	1.19	G.	6-phospho-beta-glucosidase (*arb*)
EF0361			3.34	4.03	C.	chitinase family 2 protein
EF0362			3.00	4.20	C.	chitin binding protein, putative
EF0377			1.32	1.64	O.	ankyrin repeat-containing protein
EF0378			1.33	1.57	O.	N-acyl-D-amino-acid deacylase family protein
EF0392			1.19	1	H.	hypothetical protein
EF0405			1.33	1.97	O.	hydrolase, haloacid dehalogenase-like family
EF0411			1	2.78	L.	PTS system, mannitol-specfic IIBC components
EF0456			1	1.31	L.	PTS system, mannose-specific IID component
EF0459			1	1.72	O.	glucokinase regulator-related protein, N-acetylmuramic acid-6-phosphate etherase
EF0460			1.10	1.74	H.	conserved hypothetical protein
EF0461			1.15	1.88	L.	PTS system, IIA component
EF0553			1.90	3.16	L.	PTS system, mannose-specific IID component
EF0575			1	1.38	N.	cationic ABC transporter, ATP-binding protein
EF0576			1	1.51	N.	cation ABC transporter, permease protein
EF0582			1	1.34	C.	membrane protein, putative
EF0583			1	1.53	N.	ABC transporter, ATP-binding protein/permease protein
EF0664			1.16	2.03	H.	hypothetical protein
EF0677			1.61	2.17	G.	phosphoglucomutase/phosphomannomutase family protein
EF0678			1.42	2.32	O.	acetyltransferase, GNAT family
EF0743			1	2.91	H.	hypothetical protein
EF0818			1	1.44	D.	polysaccharide lyase family protein 8
EF0877			1	1.04	O.	aldo/keto reductase family oxidoreductase
EF0938			1.41	2.71	N.	sugar ABC transporter, ATP-binding/TOBE domain protein
EF0955			1	1.51	G.	aldose 1-epimerase, putative
EF0956			1.89	3.56	G.	beta-phosphoglucomutase (*pgmB*)
EF0957			1	2.67	G.	maltose phosphorylase
EF0958			1	2.79	L.	PTS system, glucose-specific IIABC components
EF0960			1	2.35	O.	endonuclease/exonuclease/phosphatase family protein
EF1014			1	1.19	H.	hypothetical protein
EF1016			1	1.59	H.	conserved hypothetical protein
EF1025			1	1.13	O.	CBS domain protein
EF1026			1	1.41	H.	conserved hypothetical protein
EF1033			1.49	2.42	E.	6-aminohexanoate-cyclic-dimer hydrolase, putative
EF1066			1	1.78	O.	hexapeptide-repeat containing-acetyltransferase
EF1068			1.14	2.27	G.	aldose 1-epimerase (*galM*)
EF1069			1	1.67	G.	galactokinase (*galK*)
EF1157			1	1.04	I.	peptidase, M20/M25/M40 family
EF1180			1.16	1	H.	conserved hypothetical protein
EF1206			2.57	3.36	G.	malate dehydrogenase, decarboxylating
EF1214			1	1.26	G.	alpha-acetolactate decarboxylase (*budA*)
EF1225			1	1.62	B.	thiamin biosynthesis ApbE, putative
EF1237			1	1.15	H.	conserved hypothetical protein
EF1238			1	1.51	G.	glycosyl hydrolase, beta-glucosidase
EF1343			1	2.84	N.	sugar ABC transporter, permease protein
EF1344			1	3.97	N.	sugar ABC transporter, permease protein
EF1345			2.50	3.27	N.	sugar ABC transporter, sugar-binding protein
EF1347			1	3.04	G.	glycosyl hydrolase, family 13
EF1348			1	3.08	G.	glucan 1,6-alpha-glucosidase, putative
EF1349			1	3.74	G.	glycosyl hydrolase, alpha-glucosidase
EF1353			1.48	1.62	G.	pyruvate dehydrogenase complex E1 component, alpha subunit (*pdhA*)
EF1354			1.23	1.55	G.	pyruvate dehydrogenase complex, E1 component, beta subunit (*pdhB*)
EF1355			1.67	1	G.	pyruvate dehydrogenase complex E2 component, dihydrolipoamide acetyltransferase (*aceF*)
EF1356			1.20	1.47	G.	pyruvate dehydrogenase complex E3 component, dihydrolipoamide dehydrogenase (*lpdA*)
EF1358			2.84	3.61	E.	glycerol dehydrogenase (*gldA*)
EF1359			2.65	3.82	H.	conserved hypothetical protein
EF1360			2.53	1	O.	dihydroxyacetone kinase family protein
EF1361			2.70	4.30	O.	dihydroxyacetone kinase family protein
EF1368			1	1.13	H.	conserved hypothetical protein
EF1395			1	1.59	B.	molybdenum cofactor biosynthesis family protein
EF1398			1	1.88	N.	molybdenum ABC transporter, permease protein
EF1515			1	1.24	K.	transcription antiterminator, BigG family
EF1516			1.17	1.48	L.	PTS system, N-acetylglucosamine-specific IIABC components
EF1528			1	1.84	H.	hypothetical protein
EF1529			1.07	1.60	L.	PTS system, cellobiose-specific IIC component, putative
EF1535			1	2.09	H.	conserved hypothetical protein
EF1548			1	1.00	I.	ribosomal protein S1
EF1617			1	2.60	H.	conserved hypothetical protein, ethanolamine utilization protein EutQ
EF1618			1	2.45	G.	ethanolamine utilization protein EutH (*eutH*)
EF1620			1	2.88	H.	hypothetical protein
EF1621			1	1.98	H.	conserved hypothetical protein
EF1622			1	2.08	H.	conserved domain protein
EF1632			1	1.59	L.	sensor histidine kinase
EF1634			1	1.53	O.	propanediol utilization protein PduU (*eutS*)
EF1639			1	1.14	N.	iron compound ABC transporter, ATP-binding protein
EF1707			4.06	5.89	G.	glycosyl hydrolase, alpha-mannosidase
EF1708			3.50	4.80	H.	conserved hypothetical protein
EF1709			1	1.74	K.	sugar-binding transcriptional regulator, GntR family
EF1733			1	1	N.	ABC transporter, ATP-binding/permease protein, MDR family[b]
EF1800			1	1.56	H.	conserved hypothetical protein
EF1801			1.27	1.79	L.	PTS system, mannose-specific IIA component
EF1802			1	2.07	L.	PTS system, mannose-specific IID component
EF1803			1.15	1	L.	PTS system, mannose-specific IIC component
EF1806			1	1.44	G.	tagatose-6-phosphate kinase (*lacC*)

EF1817	1	1.15	I.	serine proteinase, V8 family
EF1818	1	1.01	C.	coccolysin
EF1820	1	1.00	L.	histidine kinase, (AgrC) putative"
EF1901	1.18	1.09	N.	Mn2+/Fe2+ transporter, manganese transport protein MntH
EF1912	1.38	2.31	O.	ROK family protein
EF1913	1	1.30	H.	conserved hypothetical protein TIGR00278
EF1918	1.44	1.63	H.	conserved hypothetical protein, 6-phosphogluconolactonase
EF1919	1.63	3.38	O.	acetyltransferase, GNAT family
EF1920	2.29	3.56	N.	C4-dicarboxylate anaerobic carrier, DcuC family
EF1921	1	2.36	J.	ribonucleoside hydrolase RihC
EF1927	2.43	2.56	N.	glycerol uptake facilitator protein (*glpF*)
EF1928	2.01	2.45	G.	alpha-glycerophosphate oxidase (*glpO*)
EF1929	1	2.66	G.	glycerol kinase (*glpK*)
EF1951	2.97	5.03	O.	phosphosugar-binding protein
EF1952	2.29	1	L.	PTS system, mannose-specific IID component (*mphD*)
EF2208	1	1.06	D.	phenazine biosynthesis protein PhzF family
EF2213	1.99	1	L.	PTS system, trehalose-specific IIBC components
EF2217	2.32	1	C.	alpha-1,2-mannosidase, putative
EF2220	1	1.73	H.	conserved hypothetical protein
EF2221	4.42	5.86	N.	ABC transporter, substrate-binding protein
EF2223	3.13	5.38	N.	ABC transporter, permease protein
EF2440	1.63	2.81	O.	CelC-related protein
EF2441	1	2.37	H.	conserved hypothetical protein
EF2442	1	2.53	N.	phosphate transporter family protein, PiT family
EF2559	1	2.48	G.	pyruvate flavodoxin/ferredoxin oxidoreductase family protein
EF2560	1	2.45	A.	glutamate synthase (NADPH), small chain (*gltA*)
EF2561	1	1.24	J.	ferredoxin-NADP(+) reductase subunit alpha
EF2562	1	2.07	G.	flavodoxin
EF2563	1	2.23	H.	hypothetical protein
EF2568	1.85	3.07	O.	aminotransferase, class V
EF2569	1.98	3.46	H.	conserved hypothetical protein
EF2570	1.37	2.29	O.	aldehyde oxidoreductase, putative
EF2571	1.0	1.51	H.	conserved domain protein
EF2574	1	1.21	M.	endoribonuclease L-PSP, putative
EF2711	1.42	3.08	K.	transcriptional regulator, AraC family
EF2863	3.86	5.32	G.	endo-beta-N-acetylglucosaminidase
EF2964	2.69	3.68	L.	PTS system, ascorbate-specific IIC component (*ulaA* , *sgaT*)
EF2965	1.47	1.80	L.	PTS system, ascorbate-specific IIB component
EF2966	1	1.02	K.	transcriptional regulator (formerly; transcriptional antiterminator, BglG family)
EF2975	1	1.02	H.	hypothetical protein
EF3060	2.76	3.75	O.	secreted lipase, putative
EF3061	1.64	1.92	C.	rod shape-determining protein MreD (*mreD*)
EF3062	1	1.06	C.	rod shape-determining protein MreC (*mreC*)
EF3071	1	1.17	H.	hypothetical protein
EF3082	1	1.47	N.	iron compound ABC transporter, substrate-binding protein
EF3106	1.90	2.33	N.	peptide ABC transporter, peptide-binding protein
EF3107	2.51	2.76	N.	peptide ABC transporter, permease protein
EF3108	2.48	2.71	N.	peptide ABC transporter, permease protein
EF3109	1.82	1.94	N.	peptide ABC transporter, ATP-binding protein
EF3110	1.57	2.27	N.	peptide ABC transporter, ATP-binding protein
EF3134	2.64	3.65	G.	2-dehydro-3-deoxyphosphogluconate aldolase/4-hydroxy-2-oxoglutarate aldolase (*eda-2*)
EF3135	2.30	1	G.	mannonate dehydratase, putative (*uxuA*)
EF3136	1	3.31	L.	PTS system, mannose-specific IIA component
EF3137	1.92	3.71	L.	PTS system, mannose-specific IIB component
EF3138	2.82	3.47	L.	PTS system, mannose-specific IID component
EF3139	2.34	3.06	L.	PTS system, mannose-specific IIC component
EF3140	2.75	4.07	G.	alcohol dehydrogenase, iron-containing
EF3141	1.94	2.85	O.	D-isomer specific 2-hydroxyacid dehydrogenase family protein
EF3142	1	2.58	G.	6-phosphogluconate dehydrogenase family protein
EF3144	1.50	1.92	K.	phosphosugar-binding transcriptional regulator, RpiR family
EF3146	1	1.34	H.	hypothetical protein
EF3157	4.24	4.38	G.	glycosyl hydrolase, family 65
EF3158	4.48	5.68	G.	hydrolase, haloacid dehalogenase-like family
EF3198	1.53	1.86	C.	lipoprotein, YaeC family
EF3199	1.98	1.79	N.	ABC transporter, permease protein
EF3303	1	1.02	H.	myosin-cross-reactive antigen (*mycA*)
EF3306	1.17	1	L.	PTS system, sorbitol-specific IIBC components
EF3314	1.70	2.87	C.	cell wall surface anchor family protein
EF3315	3.82	5.03	L.	triphosphoribosyl-dephospho-CoA synthase
EF3316	2.88	5.11	O.	malic enzyme family protein
EF3317	4.17	5.40	G.	oxaloacetate decarboxylase
EF3318	4.38	5.25	G.	apo-citrate lyase pyrophosphoribosyl-dephosph-CoA transferase (*citX*)
EF3319	4.22	5.44	G.	citrate lyase, alpha subunit (*citF*)
EF3320	4.91	6.26	G.	citrate lyase, beta subunit (*citE*)
EF3321	4.54	6.03	G.	citrate lyase, gamma subunit (*citD*)
EF3322	4.66	6.12	G.	citrate lyase ligase (*citC*)
EF3324	4.90	5.85	G.	sodium ion-translocating decarboxylase, beta subunit
EF3325	4.13	6.41	G.	sodium ion-translocating decarboxylase, biotin carboxyl carrier protein
EF3327	2.59	1	N.	citrate transporter (*citH*)
EF3328	1	3.68	K.	transcriptional regulator, GntR family
EFA0067	1.93	3.81	L.	PTS system, sucrose-specific IIABC components
EFA0069	1.59	2.86	G.	Sucrose-6-phosphate dehydrogenase (*scrB-2*)
EFA0070	1.32	3.03	G.	Sugar-binding transcriptional regulator (*scrR-2*)
Down-regulated				
EF0019	-2.80	1	L.	PTS system, mannose-specific IIB component (*mptB*)
EF0020	-2.75	-2.19	L.	PTS system, mannose-specific IIAB components (*mptAB*)
EF0021	1	-1.49	L.	PTS system, mannose-specific IIC component" (*mptC*)
EF0022	-1.70	-3.04	L.	PTS system, mannose-specific IID component (*mptD*)
EF0024	1	-2.09	L.	hypothetical protein (*manO*)
EF0082	-2.56	-5.55	N.	major facilitator family transporter
EF0195	-1.16	1	G.	phosphoglycerate mutase 1 (*pgm*)
EF0255	1	-1.27	G.	L-lactate dehydrogenase (*ldh-1*)
EF0635	-1.46	1	N.	amino acid permease family protein
EF0636	-1.15	1	N.	Na+/H+ antiporter (*nhaC-2*)
EF0717	-1.62	-3.42	L.	PTS system, fructose-specific IIABC components
EF0718	1	-2.78	G.	1-phosphofructokinase (*fruK-2*)
EF0719	-1.60	-3.42	K.	transcriptional regulator, DeoR family
EF0720	-1.33	-1.87	N.	voltage-gated chloride channel family protein
EF1046	1	1	G.	pyruvate kinase" (*pyk*)
EF1961	-1.11	-1.39	G.	enolase (*eno*)
EF1962	-1.04	-1.99	G.	triosephosphate isomerase (*tpiA*)
EF1963	-1.53	-2.22	G.	phosphoglycerate kinase (*pgk*)
EF1964	-1.38	-1.80	G.	glyceraldehyde 3-phosphate dehydrogenases (*gap-2*)
EF2641	-1.35	-2.77	N.	glycine betaine/L-proline ABC transporter, ATP-binding subunit
EF2642	-1.73	1	N.	glycine betaine/L-proline ABC transporter, glycine betaine/L-proline-binding/permease protein
