# Supplementary material for: Class IIa bacteriocin resistance in Enterococcus faecalis V583: The mannose PTS operon mediates global transcriptional responses
Source: BMC Microbiol. 2010 Aug 25;10:224. doi: 10.1186/1471-2180-10-224 (PMC2941500; doi:10.1186/1471-2180-10-224)
Supplement: Additional file 2 — Table A2: Summary of the putative cre-sites of regulated genes in the mutant strains. Sequence and start position of the 63 putative promoter catabolite-responsive elements of the regulated genes in the pediocin PA-1 resistant mutants, MOM1 and MOP of E. faecalis V583. [file 1471-2180-10-224-S2.DOC]

Additional File 1: Table S1.

Summary of the putative *cre*-sites of regulated genes in the mutant strains.

Sequence and start position of the 63 putative promoter catabolite-responsive elements of the regulated genes in the pediocin PA-1 resistant mutants, MOM1 and MOP of *E. faecalis* V583.

| Start | Position^a^ | Affected ORF | Gene(s) | First gene product in regulated operon or operon | Putative *cre* sequence |
| --- | --- | --- | --- | --- | --- |
| 55486 | -51 /-254 | EF0054-52, EF0055 | *dnaQ* | hypothetical protein | TTGATAGCGCTTCCT |
| 55513 | -58 /-227 | EF0054-52, EF0055 | *dnaQ* | hypothetical protein | TCGAAAGCGCTTTCT |
| 71055 | -184 /-15 | EF0068, EF0069 |  | hypothetical protein, N-acetylmannosamine-6-phosphate 2-epimerase | TTGTAATCGCTTTTAA |
| 80048 | -133 | EF0082 |  | major facilitator family transporter | TTGTAACGGATATCT |
| 106751 | -50 /-179 | EF0102, EF0103-07 |  | ArgR family transcriptional regulator, ArgR family transcriptional regulator | TGGCACCGTTTTAAA |
| 107583 | -131 | EF0104-07 | *arcA*, *argF-1*, *araC* | *arc operon* | AAGAATGCGCTTTCATA |
| 117499 | -20 | EF0114 |  | glycosy hydrolase family protein | AAGAAAGCGCATACA |
| 120275 | -34 | EF0115 |  | endoribonuclease L-PSP, putative | AATGTAAGCGGAATCAT |
| 170770 | -97 | EF0173 | *pyn* | pyrimidine-nucleoside phosphorylase | TGTTTGCGTTTTCAC |
| 229519 | -23 | EF0253 |  | aldehyde dehydrogenase | ATTGTAAGCGGATACAA |
| 232401 | -143 /-178 | EF0255, EF0256 | *ldh, pth* | L-lactate dehydrogenase, peptidyl-tRNA hydrolase | TGGTAGCTCTTACAT |
| 255755 | -167 | EF0269-71 |  | PTS system beta-glucoside-specific IIABC component | GTTGAAAGCGTTGACAT |
| 330891 | -26 /-175 | EF0362-61, EF0363 |  | chitin binding protein putative, ISEf1 transposase | TTGTATGCGCTTACA |
| 345974 | -281 | EF0377-78 |  | ankyrin repeat-containing protein | AGAAAGCGTTTTAAT |
| 362992 | -111 | EF0392 |  | hypothetical protein | ATGAAACCGATACCA |
| 377148 | -57 /-128 | EF0405, EF0406 |  | HAD superfamily hydrolase, PTS system, IIBC component | ATGTAAACGGATTCT |
| 382024 | -126 /-44 | EF0409, EF0411 |  | hypothetical protein, PTS system, mannitol-specfic IIBC components | TGGAAAGCGGATACA |
| 424601 | -130 /-17 | EF0454, EF0455 |  | hypothetical protein, PTS system, IIC component | AAGAAAACGCATTCAA |
| 592535 | -50 | EF0636 | *nhaC-2* | Na+/H+ antiporter | TTGGAAACTGAAGCA |
| 592567 | -18 | EF0636 | *nhaC-2* | Na+/H+ antiporter | AGTTTTCGCTTTCCA |
| 616102 | -2 | EF0664 |  | hypothetical protein | AATGAAAGCGGATACAG |
| 902944 | -45 /-125 | EF0938, EF0939 | *mgsA* | sugar ABC transporter ATP-binding protein, methylglyoxal synthase | AATGAAAACGCTATCTT |
| 920952 | -42 /-225 | EF0957, EF0958 |  | maltose phosphorylase, PTS system, IIABC components | ATGCAATCGGTTGCGT |
| 972102 | -130 | EF1012-16 |  | PTS system, IIB component | TGTAAGCGTTTTCTT |
| 983712 | -122 /-32 | EF1024, EF1025+26 | *ppdK* | CBS domain protein, pyruvate phosphate dikinase | ATTGTAAGCGAATGCAC |
| 993489 | -89 /-131 | EF1032, EF1033 | *drrC* | daunorubicin resistance protein, 6-aminohexanoate-cyclic-dimer hydrolase putative | ATGAAAGCGATTAAAA |
| 997586 | -60 | EF1036 |  | nucleoside diphosphate kinase | AATGAAAGCGGATACTT |
| 1040612 | -153 /-53 | EF1068, EF1069 | *galM*, *galK* | aldose 1-epimerase, galactokinase | TTGCAAATGTTTACT |
| 1126740 | -74 | EF1156+57 | *nth* | GntR family transcriptional regulator | AAGAAACCGCAATCA |
| 1147145 | -31 | EF1179+80 | *cscK* | hypothetical protein, fructokinase | ATGTATCCGCTTACTT |
| 1174105 | -38 /-94 | EF1207+06, EF1209 |  | citrate carrier protein CCS family, sensory box histidine kinase | AATGTAAACGTTTTCTG |
| 1191452 | -86 | EF1224 |  | Cro/CI family transcriptional regulator | ATGAAAGAGTTGTCT |
| 1317202 | -40 /-92 | EF1345, EF1346-49 |  | sugar ABC transporter sugar-binding protein, hypothetical protein | ATGCAACCGATTGCG |
| 1326535 | -343 | EF1353-56 | *pdhAB, aceF, lpdA* | pyruvate dehydrogenase complex E1 component, alpha subunit | ATGAAAACGCAAAGT |
| 1326673 | -205 | EF1353-56 | *pdhAB, aceF, lpdA* | pyruvate dehydrogenase complex E1 component, alpha subunit | AAGAAAACGGTTTCT |
| 1333338 | -76 /-71 | EF1358, EF1357 | *gldA* | AraC family transcriptional regulator, glycerol dehydrogenase | AAGAAAACGATACCAA |
| 1333372 | -110 /-37 | EF1358, EF1357 | *gldA* | AraC family transcriptional regulator, glycerol dehydrogenase | ATGAAAGCGTTTTAT |
| 1345439 | -71 | EF1368 |  | hypothetical protein | TTTAAAGCGTTTTCT |
| 1372026 | -24 | EF1392-95+98 | *moaC* | molybdenum cofactor biosynthesis protein MoaC | GTGTAAACGTTAACAA |
| 1489659 | -135 /-33 | EF1534, EF1535 |  | peptidyl-prolyl cis-trans isomerase cyclophilin-type, hypothetical protein | ATGTAAGCAGATACA |
| 1589608 | -132 | EF1634-17 |  | propanediol utilization protein PduU | GATGAAATCGATAACAT |
| 1657585 | -43 /-100 | EFEF1708-07, EF1709 |  | hypothetical protein, GntR family sugar-binding transcriptional regulator | ATGTAAACGCTATAA |
| 1716334 | -98 | EF1769 |  | PTS system, IIB component, putative | TTTAAAGCGGATACA |
| 1744865 | -26 | EF1800 |  | hypothetical protein | TTGAACACGCTTTCAT |
| 1749400 | -14 | EF1805-00 |  | glycosyl hydrolase family 35, PTS system IIBCDA components | ATTGAAAGCGTTTACTA |
| 1753452 | -113 | EF1809-05 | *lacC* | GntR family transcriptional regulator | ATGATAACGTTTACATT |
| 1775915 | -250 | EF1824 |  | glycosyl hydrolase | ATGAATGCGTTTTCATA |
| 1846357 | -90 /-93 | EF1901, EF1902 |  | manganese transport protein MntH, glyoxylase family protein | TTGTAAACTGTTACAT |
| 1863035 | -62 /-107 | EF1921, EF1922 |  | ribonucleoside hydrolase RihC, transcriptional regulator, LacI family/carbohydrate kinase, PfkB family protein | ATGAAAGCGCTGTTA |
| 1872750 | -21 | EF1929-27 | *glpK*, *glpF* | *glp* operon, glycerol kinase | TTTGAAATCGTTTTCTT |
| 1872861 | -132 | EF1929-27 | *glpK*, *glpF* | *glp* operon | ATTGAAAGCGTTGTCTT |
| 2126180 | -71 /-127 | EF2213, EF2214 |  | PTS system IIBC components, glyoxylase family protein | TGGCAAGCGCTTTCT |
| 2137986 | -24 | EF2223-20 |  | ABC transporter permease protein | ATGAAAACGCTATTA |
| 2478819 | -53 | EF2562 |  | flavodoxin | TTGTAACCCCTTACAT |
| 2500941 | -38 /-352 | EF2582, EF2583 |  | putative chlorohydrolase/aminohydrolase, hypothetical protein | TTGTAAGCGTTTGCT |
| 2501026 | -123 /-267 | EF2582, EF2583 |  | putative chlorohydrolase/aminohydrolase, hypothetical protein | TTGTAAGCGTTTGCTA |
| 2626254 | -122 /-23 | EF2710, EF2711 |  | amino acid permease family protein, AraC family transcriptional regulator | ATGTAAAGGCTTTCT |
| 2745434 | -30 | EF2863 |  | endo-beta-N-acetylglucosaminidase | TTTGTAAGCGCTAACAA |
| 2937980 | -121 /-145 | EF3065(62-60), EF3066 | *rpsO (mreCD), def* | ribosomal protein S15, peptide deformylase | ATGCAAGCTTAATCT |
| 3015995 | -53/-344 | EF3142-34, EF3144 |  | 6-phosphogluconate dehydrogenase-like protein, RpiR family phosphosugar-binding transcriptional regulator | AATGTAAACGATTACA |
| 3016301 | -360 /-39 | EF3142, EF3144 |  | 6-phosphogluconate dehydrogenase-like protein, RpiR family phosphosugar-binding transcriptional regulator | ATGAAAAGGCATTCAC |
| 3212039 | -358 /-29 | EF3326-10, EF3327-28 | *citCDEFX* | cit operon, citrate transporter | TTGATTAACGCTTACAA |
| 55870^b^ | -35 | EFA0067, EF0069 | *scrB-2* | sucrose-6-phosphate hydrolase, PTS system sucrose-specific IIABC components | ATTAGTAAACGTTTTCTC |

^a^The sequence start refers to nucleotide sequence in V583, and position is distance from *cre*-sequence to start codon of affected gene.

^b^In pTEF1.
